# Compressed Nonlinear Equalizers for 112-Gbps Optical Interconnects: Efficiency and Stability

**DOI:** 10.3390/s20174680

**Published:** 2020-08-19

**Authors:** Wenjia Zhang, Ling Ge, Yanci Zhang, Chenyu Liang, Zuyuan He

**Affiliations:** State Key Laboratory of Advanced Optical Communication Systems and Networks, Shanghai Jiao Tong University, Shanghai 200240, China; mrgreen@sjtu.edu.cn (L.G.); zhangyanci@sjtu.edu.cn (Y.Z.); lcy1993@sjtu.edu.cn (C.L.); zuyuanhe@sjtu.edu.cn (Z.H.)

**Keywords:** VCSEL, neural network-based equalization, Volterra series-based equalization

## Abstract

Low-complexity nonlinear equalization is critical for reliable high-speed short-reach optical interconnects. In this paper, we compare the complexity, efficiency and stability performance of pruned Volterra series-based equalization (VE) and neural network-based equalization (NNE) for 112 Gbps vertical cavity surface emitting laser (VCSEL) enabled optical interconnects. The design space of nonlinear equalizers and their pruning algorithms are carefully investigated to reveal fundamental reasons of powerful nonlinear compensation capability and restriction factors of efficiency and stability. The experimental results show that NNE has more than one order of magnitude bit error rate (BER) advantage over VE at the same computation complexity and pruned NNE has around 50% lower computation complexity compared to VE at the same BER level. Moreover, VE shows serious performance instability due to its intricate structure when communication channel conditions become tough. Moreover, pruned VE presents more consistent equalization performance within varying bias values than NNE.

## 1. Introduction

Recent decades have witnessed the explosion of data traffic especially in regional spaces like data centers and supercomputers. The hardware infrastructure for supporting such massive connectivity has been turning to optical components and fibers even over a distance less than 100 m. The data rate of optical interconnects have exceeded 100 Gbps per lambda in commercialized products and will soon upgrade to 200 Gbps per lambda for 800 Gbps or 1 Tbps optical module [1]. Vertical cavity surface emitting laser (VCSEL)-based optical interconnect is a typical and competitive candidate because of interesting features of low cost and power consumption [2]. To meet capacity, cost and power consumption requirements, intensity modulation and direct detection (IM-DD) with more advanced modulations like four- or eight-level pulse amplitude modulation (PAM4/8) [3], carrier-less amplitude phase modulation (CAP) [4] and discrete multi-tone modulation (DMT) [5] has been proposed for 100/200 Gbps per lambda optical interconnects, in which PAM4 has become a widely accepted modulation format for 400G products. It is of necessity rather than an option to employ advanced modulations for improving spectral efficiency due to severe bandwidth limitation and noise accumulation of devices and channels. However, advanced modulations, though mitigating the system requirement on frequency response of critical devices, will also bring serious modulation non-linearity [6,7], reduced signal–noise ratio (SNR), and level-dependent noise accumulation [8]. In particular, VCSEL-based interconnect solution confronts large challenges to realize reliable 100/200 Gbps per lambda communications with advanced modulations mostly because of bandwidth limitation [9] and complex impairment combination of relative intensity noise (RIN) [10], mode partition noise (MPN) [11] and fiber mode dispersion. The complicated signal distortions in time, amplitude and frequency domain need advanced nonlinear equalization for compensation and adaption [3,6,8,11,12,13,14].

Advanced digital signal processing (DSP) technologies-based nonlinear equalization, such as Volterra series-based equalization (VE) [3,15,16,17,18] and neural network-based equalization (NNE) [12,19,20,21,22], have been proposed to realize powerful nonlinear compensation capability in the VCSEL enabled optical interconnect. However, high computation complexity of these nonlinear equalization hinders their practical implementation in the cost and power-sensitive module. Fortunately, it has been proved that there are various design spaces for nonlinear equalization to reduce computation complexity without sacrificing communication quality [3,12]. Therefore, only by marrying strong algorithms with limited computing and storage resources of underlying hardware, nonlinear equalization can find their values in the next wave of hardware evolution for a high-end optical module. There are various low-complexity designs of VE and NNE in the literature. In [23], coefficients in Volterra series that are not on the diagonal have been intentionally abandoned to construct a memory polynomial Volterra equalizer. However, this approach is not able to adapt to various transmission conditions since that simplification process runs before training. Moreover, in [12,17,24], the authors employ an approach of weight-pruning and retraining to reach a sparse but optimized equalizer structure for VE or NNE. Additionally, more aggressive methods [3,16,18] by introducing $\ell_{0}$ and $\ell_{1}$-regularization penalty term into original cost function for pruning Volterra nonlinear equalizer are proposed to force large amount of coefficients to zeros, demonstrating a over 93% complexity reduction in the 112 Gbps VCSEL-based optical interconnects. However, equalization architectures for Volterra series and neural network are fundamentally different in the nonlinear construction. In addition, there is tremendous difference in the growth pattern of computation complexity with construction parameters. So, pruning strategies should be carefully designed for two equalization architectures for exploiting best potentials. Questions are raised on which nonlinear equalization prevails over others of realizing lowest complexity equalization while keeping the reliable transmission performance [25]. More importantly, it is still unclear the window of key parameter variation for a working channel that equalization performance gain can keep consistent.

In this paper, we compare the efficiency and stability of a pruned three-layer neural network-based equalizer and a pruned three-order Volterra series-based equalizer for VCSEL enabled 112 Gbps optical interconnects. A threshold-based pruning and retraining algorithm, which is proposed in our previous publications [3,12], is used to compare the pruning efficiency of nonlinear equalizers. In this comparison study, we extend the conference paper [25] by elaborating design space of nonlinear equalizers, including engineering parameters on memory length, neural network layer and activation function, in order to reveal fundamental reasons of powerful nonlinear compensation capability and restriction factors of efficiency and stability. In addition, signaling performance under different bias voltages for a VCSEL is evaluated by using pruned VE and pruned NNE, respectively.

## 2. Principle of Pruned Nonlinear Equalizers

### 2.1. Volterra Series and Neural Network-Based Equalizer

The mathematical expression of *P*-order VE with memory length of $M_{r}$ can be expressed as Equation (1),

(1) $$y\left(k\right)=W_{dc}+\sum_{r=1}^{P}\sum_{k_{1}=0}^{M_{r}-1}\cdots \sum_{k_{r}=k_{r-1}}^{M_{r}-1}W_{r}\left(k_{1},k_{2},\ldots ,k_{r}\right)\cdot x\left(k-k_{1}\right)\cdots x\left(k-k_{r}\right).$$

where x(k) is $k^{th}$ sampled data from received signals and y(k) is output data through equalization. $W_{r}$ is the $r^{th}$-order Volterra kernel. $W_{dc}$ is responsible for DC component, which is not included in the final model of an AC-coupled circuit. This model presented in Equation (1) can approximate any nonlinear system in theory. However, it is physically impossible in many cases due to high computation complexity. As low-order part of Volterra series model indicates most features of nonlinear system, approximation approaches are often used to truncate theoretically infinitely long expressions.

It can be seen from Equation (1), the number of coefficients, indicating computation burden of multiplication and addition, will grow very fast with increase of *P* and memory length $M_{r}$. It has been known that multiplication contributes most of computation resources compared to addition and will be main reason of complexity effect of pruning algorithms [18]. Therefore, computation complexity for a *P*-order VE with memory length of $M_{r}$ can be defined as the number of multiplication operations [18] and expressed as Equation (2),

(2) $$Complexity=M_{1}+M_{2}\left(M_{2}+1\right)+M_{3}\left(M_{3}+1\right)\left(M_{3}+2\right)/2+\cdots$$

According to [3,26], a three-order structure is sufficient for short-reach optical interconnects. By expanding Equation (1), a three-order VE, named by $VE(M_{1},M_{2},M_{3})$ where $M_{1},M_{2},M_{3}$ are memory lengths of first, second and third order of VE, is described by

(3) $$\begin{array}{cc} \; & y\left(k\right)=\sum_{k_{1}=0}^{M_{1}-1}w_{1}\left(k_{1}\right)x\left(k-k_{1}\right)\\ \; & +\sum_{k_{1}=0}^{M_{2}-1}\sum_{k_{2}=k_{1}}^{M_{2}-1}w_{2}\left(k_{1},k_{2}\right)x\left(k-k_{1}\right)x\left(k-k_{2}\right)\\ \; & +\sum_{k_{1}=0}^{M_{3}-1}\sum_{k_{2}=k_{1}}^{M_{3}-1}\sum_{k_{3}=k_{2}}^{M_{3}-1}w_{3}\left(k_{1},k_{2},k_{3}\right)x\left(k-k_{1}\right)x\left(k-k_{2}\right)x\left(k-k_{3}\right). \end{array}$$

**Figure 1:**
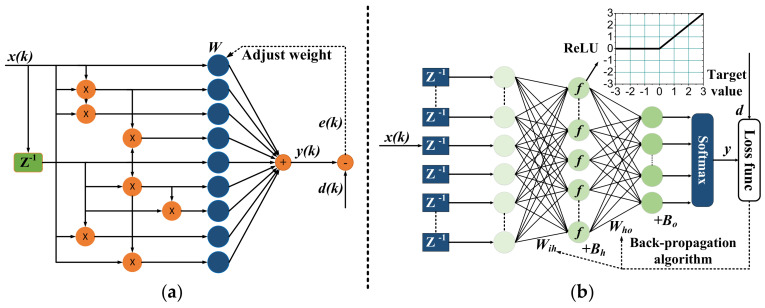
The structure of (**a**) three-order VE(2,2,2) and (**b**) three-layer NNE.

A basic VE(2,2,2) structure with 9 coefficients is shown in Figure 1a. In this work, a three-order VE (P=3) is applied for further experimental investigation.

Artificial neural networks have been widely deployed in various areas such as image classification and natural language processing, showing much better performance than traditional algorithms. Recently, it has been intensively researched in the field of optical communication, in which nonlinear equalization is one of hottest topic [13]. To equalize PAM-N communication signals, the received analog signals are taken to a neural network with N possible outputs, representing N levels. The signal stream is first delayed through a delay array, and then input into an input layer with designed neuron number. The numbers of input neuron and layer number are also of great significance to balance the equalization performance and computation complexity. According to universal approximation theorem, a three-layer neural network will provide strong nonlinear equalization capability for the communication systems.

The mathematical formula of NNE process can be expressed as Equation (4),

(4) $$y=argmax[softmax\left(f\left(x^{T}\left(k\right)\times W_{ih}+B_{h}\right)\times W_{ho}+B_{o}\right)]$$

where *x(k)* is a sampled signal sequence, $W_{ih}$ and $W_{ho}$ are weight matrices of input layer to hidden layer and hidden layer to output layer respectively. $B_{h}$ and $B_{o}$ are bias vectors of hidden layer and output layer. *f* means activation function of hidden layer. Rectified linear unit (ReLU), tangent hyperbolic (Tanh) and sigmoid functions are considered in this work. Function softmax(·) is used to convert the results of output layer to probability distribution for each class. Finally, argmax[·] is decision function, which returns an index of the maximum value of output probability vector. *y* is final result indicating a specific symbol of information. Loss functions of ANN, including mean square error (MSE) loss function, logarithmic loss function and cross-entropy loss function, are important for training model and final performance. The MSE loss function usually shows slow convergence and is seldom used in classification task. In this work, back propagation (BP) algorithm combined with cross-entropy loss function are used to train NNE to achieve better classification performance.

The structure of the 3-layer NNE is shown in Figure 1b, where $NNE(N_{1},N_{2},N_{3})$ represents a 3-layer NNE with $N_{1},N_{2},N_{3}$ as neuron number of input, hidden and output layer. Similar to VE, computation complexity for NNE is defined as number of multiplication operation as calculated in Equation (5),

(5) $$Complexity=N_{1}\times N_{2}+N_{2}\times N_{3}+N_{3}\times N_{4}+\cdots$$

### 2.2. Pruning Algorithm

It has been proved that most of multiplication operations in the nonlinear equalizers are redundant [23] as either weight value is small or connections in network level contribute very little to final results. Therefore, there is possibility of best pruning algorithm that can realize a lightest equalizer without sacrificing transmission performance. The choice of strategy to prune the complex networks as shown in Figure 1 become very important to reduce computation complexity for each architecture while maintaining equalization performance. We proposed a threshold-based pruning and retaining approach for equalizers, where coefficients after initial training are intentionally discarded based on a threshold and damages caused by pruning process are recovered as much as possible through retraining [3,12]. The pruning process can be easily expressed as Equation (6), where S(·) represents weight setting and *T* is threshold. With weight value setting to zero, connections relating to this value is cut in these complex networks. Due to different features of two architectures, there also needs special design considerations for each equalizer. For VE, second- and third-order coefficients are pruned because we find linear terms only occupy a small amount of computation complexity while contribute most on the performance gain due to bandwidth limitation of the experimental system. For NNE, all weights both from input layer to hidden layer and hidden layer to output layer are run for pruning based on the threshold. An iterative pruning process is developed to achieve a more efficient network structure.

(6) S(W)=0,whenS(·)<T.

## 3. Experiment and Results

### 3.1. Experiment Setup

**Figure 2:**
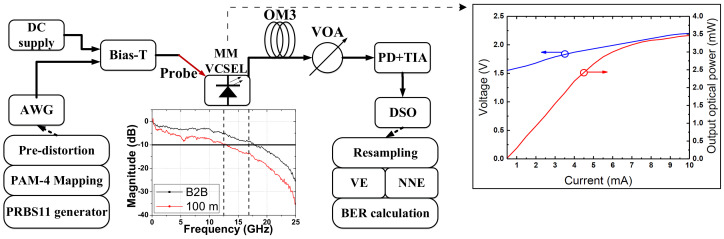
Experimental setup.

Figure 2 shows experimental setup for 112 Gbps optical interconnects. In the transmitter, 112 Gbps pseudo-random binary sequence (PRBS)-11 PAM-4 signals are generated by an arbitrary waveform generator (AWG), which is Keysight M8195A with 64-GSa/s sampling rate and 25-GHz 3dB bandwidth. Please noted that the waveform generator is bandwidth limited for generating 112 Gbps PAM4 signals, but this inherent bandwidth limitation can be mitigated by pre-distortion and equalization. PRBS-11 is used due to memory limitation of our AWG. High-speed data-streaming combined with DC bias are applied on an 850 nm multimode VCSEL bare die through a high-speed probe. Light-current-voltage (LIV) curve and 3-dB bandwidth of VCSEL can be found in the inset of the Figure 2. Lights from VCSEL are coupled into a 100 m OM3 multimode fiber through a three-dimension coupling station. Through a variable optical attenuator (VOA), lights are detected by a photodetector (PD, New Focus 1484-A-50) integrated with a transimpedance amplifier (TIA). At the receiver, detected signals are sampled and captured by a real-time digital storage oscilloscope (DSO, Keysight DSOZ592A) with 160-GSa/s sampling rate and 59-GHz analog bandwidth for offline digital signal processing. The captured data are re-sampled to one sample per symbol, followed by nonlinear equalization and bit error rate (BER) calculation.

To avoid the risk of performance over-estimation from nonlinear equalization, we randomly disrupt the signals to create a sequence without periodicity before the received signals are fed into equalizer and conducted mini-batch training [12]. After such process, a disrupted sequence, with an ultra-long unrepeated pattern, is used to train equalizer, which enables equalizer to learn system characteristics instead of generation rules of PRBS.

### 3.2. Efficiency Comparison

**Figure 3:**
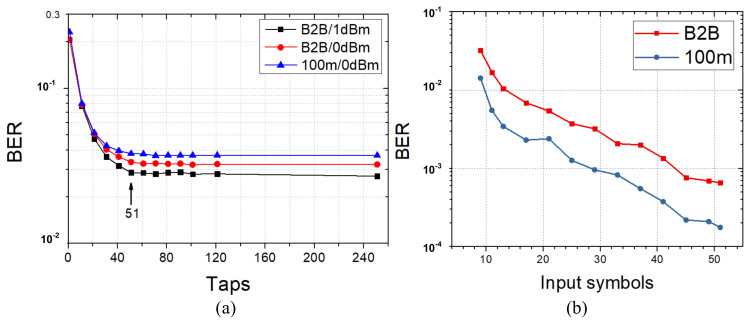
(**a**) BER versus memory length of first-order VE with received optical power of 1 dBm and 0 dBm (B2B) and 0 dBm (100 m); (**b**) BER versus input symbol number with 51 hidden neurons and three layers and received optical power of 1 dBm.

**Figure 4:**
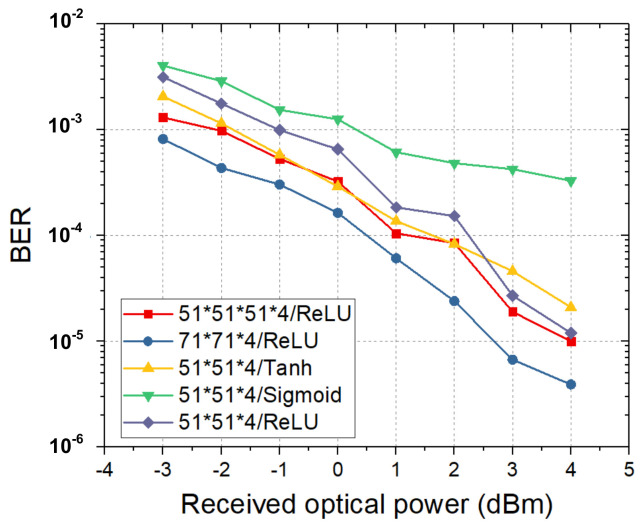
Impact of activation function and layer number of NNE on equalization performance.

First, memory length should be carefully tuned for VE and NNE to better understand the efficiency of pruning algorithms. If these parameters are intentionally set with large numbers, complexity reduction ratio will be exaggerated through pruning due to expanded redundancy, which is not fair for comparison. Therefore, we have tuned memory length of first order in VE by setting high-order memory length to zeros. Figure 3a shows that BER keeps stable when memory length of this order is from 51 to 251 for 100 Gbps PAM4 transmission, which is similar for back-to-back (B2B) and 100 m transmission with various received optical powers. Therefore, 51 is set for the first-order memory length. For nonlinear terms selection, second- and third-order memory lengths are increased simultaneously while trying to make their coefficient number in a similar level in order to balance performance gain introduced by second- and third-order term. For NNE, we fix the number of hidden layer neurons as 51 and only change the number of input symbols from 9 to 51. Figure 3b shows BER is reduced with increasing input symbol. The smooth BER change, without large-scale jump between any adjacent two points, also indicates that NNE in our experiment does not obtain over-estimation gain thanks to the use of randomization process, since that over-estimation problem for NNE will lead to stair-like curve of BER versus input symbol number [27]. Moreover, design space for NNE also includes activation function and layer number, which influence equalization performance. As shown in Figure 4, ReLU and Tanh activation function show better BER performance than Sigmoid. Tanh has similar BER with ReLU when optical power is smaller than 2-dBm while ReLU will achieve better performance when received optical power is more than 2-dBm. We can also learn from Figure 4 that BER performance can be slightly improved by extending the layer number of NNE from three to four with the same activation function of ReLU through comparing a three-layer NNE(51,51,4) with a four-layer NNE(51,51,51,4). However, more BER performance improvement can be obtained by increasing input neuron number to 71 by comparing three-layer NNE(51,51,4) with NNE(71,71,4).

**Figure 5:**
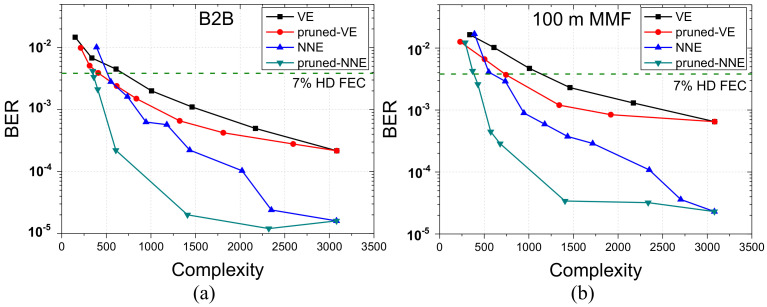
The efficiency comparison for (**a**) B2B case and (**b**) 100 m MMF case at 100 Gbps PAM-4 with pre-distortion.

Second, 100 Gbps PAM4 signaling at a typical 1-dBm received optical power is used for comparing efficiency of VE and NNE and their pruning version. Figure 5 shows the experimental results at B2B and 100 m MMF cases. We carefully set the initial equalizer configurations of VE(51,31,15) and NNE(51,56,4) with the initial complexity 3083 for VE and 3080 for NNE in order to balance the initial complexity of VE and NNE for fair comparison. As we can see from Figure 5, NNE presents more than one order of magnitude BER advantage over VE for both B2B and 100 m MMF cases at the same computation complexity. By using pruning algorithms, lower computation complexity could be achieved for both VE and NNE. At 7% HD FEC limit of 100 m MMF case, complexity values are 1144 and 603 for general VE and NNE and is reduced to 743 and 386 with pruning algorithms, in which pruned NNE shows 48% complexity performance improvement than pruned VE.

**Figure 6:**
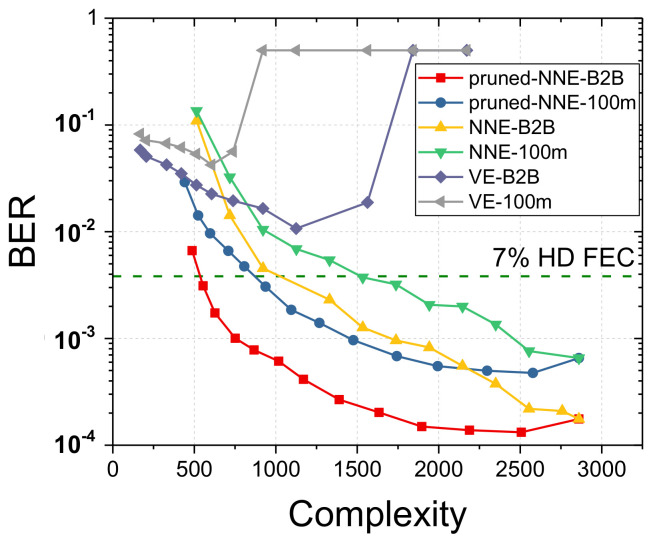
The efficiency comparison at 112 Gbps PAM-4 without pre-distortion.

Third, in order to better understand behavior of VE and NNE, we increase the data rate to 112 Gbps and eliminate the pre-distortion process for pressuring more on the bandwidth limitation as indicated in the inset of Figure 1. Figure 6 illustrates experimental results with received optical power of 0-dBm. Noted that the computation complexity of equalizers without pruning process is changing with memory lengths of equalization architecture. From the Figure 6, VE shows serious numerical instability due to its intricate structure. With memory length increasing, BER performance powered by VE shows a slow reduce and even starts to increase until misconvergence with BER of 0.5. On the other hand, NNE is still able to achieve remarkable equalization performance for B2B and 100 m cases. Therefore, NNE has significantly more robust performance than VE in a harsh link situation. With pruning algorithms, NNE attains around 50% complexity reduction compared to conventional NNE at the FEC limit of BER with 3.8 × 10${}^{-3}$. In addition, at the same complexity, equalizer without pruning algorithms shows worse BER performance due to reduced memory length. From Figure 6, we also learn that VE outperforms NNE when complexity is lower than 800 since that VE can always maintain the least linear equalizer. However, the performance of NNE, on the other hand, will decline rapidly after large-scale pruning, as NNE fails to have the property of separate order like VE.

### 3.3. Stability Comparison

**Figure 7:**
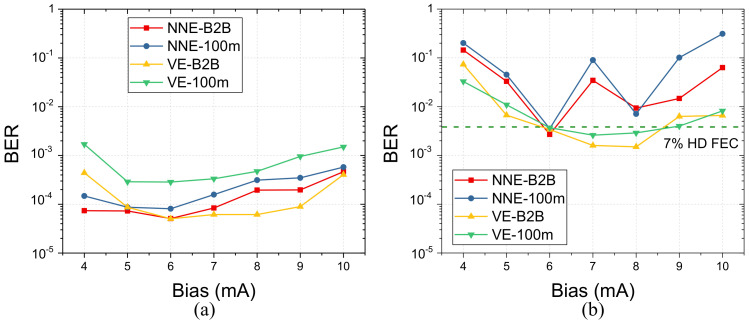
BER versus VCSEL DC bias for (**a**) VE and NNE (**b**) Pruned VE and NNE.

Performance stability for equalization is of great significance in the real application where output optical power or 3-dB bandwidth of critical devices in the optical interconnect will change within a life-cycle because of component aging or ambient temperature fluctuation. It is likely that performance stability will become worse with pruning algorithms since that the redundancy of connections in the equalization will be useful in changing scenarios. To verify the stability of pruning algorithm with changing link conditions, we choose DC bias of VCSEL as a key parameter for further evaluation because DC bias variation of VCSEL will not only change the output power but also bandwidth of directly modulated laser. The transmission experiment is run with a 100 Gbps PAM4 pre-distorted link. We set VE(51,23,11) with complexity of 1461 and NNE(31,41,4) with complexity of 1435, respectively. BER performance under different bias is shown in Figure 7. The BER bathtub curve indicates that equalization performance variation, instead of bandwidth variation with bias current, is the main reason of BER deviation. First, BER is calculated as function of bias for NNE and VE with B2B and 100 m condition. Figure 7a shows stable BER level with bias from 4 mA to 10 mA. 6 mA, the optimal operating point of VCSEL, is chosen as a benchmark point. Second, pruning algorithms for VE and NNE are running and optimized at the bias of 6 mA. The same pruned equalizers are then applied to data received with a different bias current for VCSEL. As we can see from Figure 7b, the pruned VE optimized for 6 mA bias also shows excellent tolerance for bias from 7 mA to 9 mA. This means that sparse VE structure has consistent equalization performance within a bias range of 2.5 mA in this experiment. However, this is not case for NNE. Pruned NNE, optimized using data of 6 mA bias, seems less effective and more fluctuating for cases with a different bias. The experimental results in Figure 7b of fluctuation BER with pruned NNE shows that the iterative pruning and retraining approach has broken the equalization resilience due to reduced connections between layers.

## 4. Conclusions

In this paper, we compare the efficiency and stability performance of pruned Volterra series-based equalization and neural network-based equalization for 112 Gbps VCSEL enabled optical interconnects. From the 112 Gbps PAM4 transmission experiment, we can conclude that NNE has more than one order of magnitude BER advantage over VE at the same computation complexity. By using pruning algorithms, NNE has around 50% lower computation complexity compared to VE at the same BER level. Moreover, when communication channel condition become tough, VE shows serious performance instability due to its intricate structure. Through performance stability evaluation by changing bias current of a VCSEL, pruned VE shows more consistent equalization performance than NNE.

## Abbreviations

The following abbreviations are used in this manuscript:

VE	Volterra series-based equalization
NNE	Neural network-based equalization
VCSEL	Vertical cavity surface emitting laser
BER	Bit error rate
IM-DD	Intensity modulation and direct detection
PAM	Pulse amplitude modulation
CAP	Carrier-less amplitude phase modulation
DMT	Discrete multi-tone modulation
SNR	Signal–noise ratio
RIN	Relative intensity noise
MPN	Mode partition noise
DSP	Digital signal processing
PRBS	Pseudo-random binary sequence
VOA	Variable optical attenuator
PD	Photodetector
TIA	Transimpedance amplifier
DSO	Digital storage oscilloscope
B2B	Back-to-back
Tanh	Tangent hyperbolic
ReLU	Rectified linear unit
